# *ABCC5* Transporter is a Novel Type 2 Diabetes Susceptibility Gene in European and African American Populations

**DOI:** 10.1111/ahg.12072

**Published:** 2014-08-12

**Authors:** Kenan Direk, Winston Lau, Kerrin S Small, Nikolas Maniatis, Toby Andrew

**Affiliations:** 1 Department of Twin Research and Genetic Epidemiology, King's College London, School of MedicineLondon, UK; 2 Department of Genetics, Evolution and Environment, University College LondonLondon, UK; 3 Department of Genomics of Common Disease, Imperial CollegeLondon, UK

**Keywords:** Type 2 diabetes, genetic maps, eQTL, candidate gene

## Abstract

Numerous functional studies have implicated *PARL* in relation to type 2 diabetes (T2D). We hypothesised that conflicting human association studies may be due to neighbouring causal variants being in linkage disequilibrium (LD) with *PARL*. We conducted a comprehensive candidate gene study of the extended LD genomic region that includes *PARL* and transporter *ABCC5* using three data sets (two European and one African American), in relation to healthy glycaemic variation, visceral fat accumulation and T2D disease. We observed no evidence for previously reported T2D association with Val262Leu or *PARL* using array and fine-map genomic and expression data. By contrast, we observed strong evidence of T2D association with *ABCC5* (intron 26) for European and African American samples (*P* = 3E−07) and with *ABCC5* adipose expression in Europeans [odds ratio (OR) = 3.8, *P* = 2E−04]. The genomic location estimate for the *ABCC5* functional variant, associated with all phenotypes and expression data (*P* = 1E−11), was identical for all samples (at Chr3q 185,136 kb B36), indicating that the risk variant is an expression quantitative trait locus (eQTL) with increased expression conferring risk of disease. That the association with T2D is observed in populations of disparate ancestry suggests the variant is a ubiquitous risk factor for T2D.

## Introduction

Genome-wide association (GWA) studies have identified over 60 loci that are associated with type 2 diabetes (T2D) (Morris et al., 2012; Visscher et al., 2012) consistent with the theory of polygenic inheritance (Clayton, 2009; Yang et al., 2010; Visscher et al., 2012). One candidate gene not previously identified in human GWA studies is mitochondrial gene *PARL*, located at 3q27 with a documented functional, but controversial role in the development of T2D (Walder et al., 2005; Fawcett et al., 2006; Powell et al., 2008; Hatunic et al., 2009; Tang et al., 2009; Civitarese et al., 2010). There is also replicated evidence of linkage to the 3q26-29 locus region for glycaemic traits (Kissebah et al., 2000), obesity (El-Sayed Moustafa & Froguel, 2013) and T2D (Hegele et al., 1999; Vionnet et al., 2000; Francke et al., 2001; Busfield et al., 2002; Mori et al., 2002; Guan et al., 2008), but these studies potentially implicate many genes in the region (Pritchard & Cox, 2002).

Walder et al. (2005) demonstrated that skeletal muscle *PARL* expression is reduced in insulin resistant and T2D-induced desert sand rats, but could be restored again by increased exercise. The study also showed a positive linear correlation between *PARL* mRNA levels and insulin sensitivity in humans and a nonsynonymous single nucleotide polymorphism or SNP (Val262Leu; rs3732581) in exon 7 of *PARL* to be associated with fasting insulin. While there is suggestive evidence of association with earlier age of T2D diagnosis (Hatunic et al., 2009), the SNP-insulin association has not subsequently been replicated (Fawcett et al., 2006; Powell et al., 2008; Hatunic et al., 2009). Other studies have also shown reduced *PARL* expression and mitochondrial function to be associated with T2D and ageing (Tang et al., 2009; Civitarese et al., 2010; Curran et al., 2010).

Despite such functional evidence, *PARL* (and genes in the immediate vicinity of *PARL*) have not been implicated with T2D in the most recent association meta-analysis of 27 case-control cohorts totalling 22,669 cases and 58,119 controls of primarily European descent or with fasting insulin and glucose levels for up to 133,010 nondiabetic individuals (Morris et al., 2012).

The aims of this study are: (1) to assess if the previously reported *PARL* missense Val262Leu polymorphism (not genotyped on commercial arrays) is associated with population fasting plasma insulin levels and (2) to conduct a comprehensive T2D candidate gene association and functional expression study of the linkage disequilibrium (LD) region (approximately 185,000–185,250 kb, Build 36) that includes *PARL* and neighbouring *ABCC5*.

## Methods

### Subjects and Data

To address these aims, we investigated the gene region by requesting the genotype and phenotype data from two publicly available data sets and collected additional fine map and expression data from TwinsUK, since our method requires original, rather than summary published data. All genomic data were screened for quality control and tested for population stratification using standard procedures (Balding, 2006) for the following samples:

1. The Wellcome Trust Case Control Consortium (WTCCC, phase I) T2D cases (*n* = 1926) and controls (*n* = 2938), genotyped using the Affymetrix GeneChip 500K chip (Wellcome Trust Case Control Consortium, 2007).
2. The African American (AA) T2D case-control data were obtained from the National Institute for Diabetes and Digestive and Kidney disease (NIDDK) with cases (*n* = 1033, controls = 971) affected by T2D and end-stage renal disease (ESRD) recruited from dialysis centres in North Carolina and neighbouring states (Palmer et al., 2012). Genotyping was performed on the Affymetrix Genome-wide Human SNP array 6.0 (∼1 million SNPs).
3. A population-based sample of European twins (TwinsUK) (Moayyeri et al., 2013) using most recent measure of fasting plasma insulin (pmol/l) and glucose (mmol/l), a validated Dual-energy X-ray absorptiometry (DEXA) based measure of visceral fat (Direk et al., 2013) and self-reported T2D. Ten-hour fasting plasma insulin measures were taken using a chemiluminesence assay (Roche 2010 analyser, pmol/ml). Glucose was measured using a Roche P800 modular system. Fasting glucose and insulin levels were mean-corrected for year of visit, age and sex (*R*^2^ = 0.18 and *R*^2^ = 0.07, respectively). We used the quantile normalised fasting plasma insulin: glucose ratio (IGR), in order to have one summary glucose homeostasis measure for analysis that was comparable with previous studies (Kissebah et al., 2000). Homeostasis Model Assessment 2 (HOMA2) values were also calculated (Levy et al., 1998) to assess beta cell function and insulin sensitivity in relation to adipose gene expression results. T2D status for TwinsUK data was ascertained via an online questionnaire, asking if a physician had ever diagnosed the individual with the condition, with approximately 5% identified as T2D (*n* = 256/5616 respondents).

We used genomic TwinsUK data measured using the Illumina HumanHap 610 Quad 610K array. The Val262Leu (rs3732581) SNP was not included on the 610K commercial array (*n* = 2300) and was therefore genotyped for a total of 3087 twin samples. For TwinsUK samples with available Illumina 610K SNP data, an additional 26 fine-map SNPs were genotyped across the analytical window with a wide range of minor allele frequencies (MAF ≥ 0.05) to improve fine-mapping genotype coverage for the *PARL* and *ABCC5* genes. Additional markers were selected using highly informative European LD genetic maps generated using publically available HapMap CEU 2.5 million SNP data (see LD maps) on the basis of where gaps on the 610K array were identified in relation to the genetic map.

Subcutaneous adipose fat, lymphoblastoid cell line (LCL) and skin messenger RNA levels were measured using the Illumina expression array HumanHT-12 version 3 for 821 twins by the MuTHER consortium with data generation and normalisation methods described elsewhere (Grundberg et al., 2012). Table S1 lists the six expression probe locations for *PARL* and *ABCC5*, with probes marking different isoforms for each of these genes. Only adipose tissue probes were used for the expression analysis, as we reasoned LCL and skin expression are unlikely to be relevant to the aetiology of T2D.

### Statistical Genetic Methods

#### Biometric model

For the candidate marker Val262Leu test of association, fasting insulin, glucose, IGR and T2D were regressed upon the Val262Leu SNP (rs3732581 G/G was coded as 0, G/C as 1 and C/C as 2) using mixed linear regression methods. A previously reported interaction model (Walder et al., 2005) testing for association between fasting insulin levels and age stratified by Val262Leu genotypes was also assessed.

#### LD maps and genetic association

The association analyses for this study utilise high-resolution genetic maps with locations expressed in LD Units (LDU map), which were recently also used to identify 200 genes for Crohn's disease (Elding et al., 2013). The LDU map and association mapping method are both based upon the Malécot model of decline of association with genetic distance in LDU (Maniatis et al., 2002; Wellcome Trust Case Control Consortium, 2007). Once the population-specific genetic maps have been estimated, an extension of the Malécot model can then be used to implement a multimarker test of association (Maniatis, 2007) utilising information from all markers in a region simultaneously to obtain a location estimate for the functional common variant (*Ŝ*) associated with the phenotype (±standard error of the genetic location). For this study, a physical analytical window of 800 kb (184,743–185,548 kb) was used that corresponds to approximately 10 LDU on the genetic map for the European samples and 17 LDU for the AAs. The 800 kb analytical window included a total of 22 genes (*KLHL6*, *KLHL24*, *YEATS2*, *MAP6D1*, *PARL*, *ABCC5*, *HTR3D*, *HTR3E*, *EIF2B5*, *DVL3*, *AP2M1*, *ABCF3*, *VWA5B2*, *MIR1224*, *ALG3*, *ECE2*, *CAMK2N2*, *PSMD2*, *EIF4G1*, *SNORD66*, *FAM131A* and *CLCN2*) located within this region, with the entire region assessed as part of one single Malécot test of association.

The construction of the LDU maps using the HapMap Phase II data and association mapping methods used for this study are described in detail elsewhere (Collins, 2007). All analyses used genomic human reference sequence March 2006 (National Center for Biotechnology Information Build 36.1, hg18).

#### eQTL regression analyses

For each expression probe (three per gene), we tested for evidence of regulatory eQTLs for *PARL* and *ABCC5* adipose tissue expression using the same Malécot modelling approach and analytical window as described above, controlling for recorded experimental batch effects by analysing regression residuals. Mixed linear regression models were also used to test if the phenotypes of insulin resistance (IGR), visceral fat accumulation and T2D were associated with adipose gene expression for the twin samples.

#### Significance thresholds

Despite being a candidate gene association study, we used a stringent genome-wide significance threshold of 1E−05 for the genetic association analyses. This conservative threshold corresponds to independent tests for 4800 analytical LDU windows that cover the entire human genome (≈0.05/5000; *α* = 1E−05) (Elding et al., 2013).

For the phenotype-expression association analyses, we used regression methods (Camp & Farnham, 2001) to estimate that the six probes correspond to the equivalent of four independent tests and that the five T2D-related phenotypes correspond to three independent tests due to the correlation structure between them. Therefore, for these analyses we used a conservative Bonferroni correction of *α* = 1E−03 (≈0.05/(3×4)).

## Results

### Val262Leu (rs3732581, *PARL* Exon 7) as a Candidate SNP for Insulin Resistance and T2D

Regression models confirmed a strong association between risk of insulin resistance, T2D and age with both fasting insulin levels (0.12 pmol/l per year) and risk of T2D [odds ratio (OR) = 1.04 per year] increasing with age (Table 1, Panel A). By contrast, Val262Leu showed no evidence of association (unadjusted or adjusted for age) with fasting plasma insulin levels (*P* = 0.95) or self-reported T2D (*P* = 0.10). We also assessed a previously suggested model by Walder et al. (2005) for an age-by-Val262Leu genotype interaction effect (Table 1, Panel B) for fasting insulin and T2D. Although both genotype groups showed a positive association between fasting insulin levels and age and T2D and age, GG individuals showed no evidence of stronger association between phenotype and age compared to CC/GC individuals, with a Wald *χ*^2^_1_ of 0.14 (*P* = 0.71) and 0.74 (*P* = 0.39) for fasting insulin and T2D, respectively (Table 1).

**Table 1:** Nonsynonymous SNP rs3732581 (Val262Leu) test of association with fasting plasma insulin levels and type 2 diabetes (T2D) for TwinsUK data

	Fasting insulin			T2D		
		
	Beta	SE	*P*-value	OR	SE	*P-*value
Panel A: Main effect model						
rs3732581	−0.17	0.56	0.95	0.82	0.12	0.10
Age_ALL_	0.12	3.E−02	1.E−04	1.04	0.01	3.E−08
Panel B: Interaction model (SNP-Age)						
Age_GG_	0.13	0.06	0.03	1.03	0.01	2.E−07
Age_GC/CC_	0.12	0.04	1.E−03	1.05	0.01	3.E−09

**Panel A (Main effect model)**: association between phenotype and SNP rs3732581 adjusted for age. Increased fasting insulin levels and risk of T2D are positively associated with age, but are not associated with Val262Leu. **Panel B (Interaction model)**: association between phenotype and age stratified by SNP using an interaction model (adapted from Walder et al., 2005). For the TwinsUK data, the postestimation Wald test contrasting two regression coefficient interaction terms provides no evidence that association between phenotype and age differs between homozygous GG and GC/CC individuals (i.e. *β*_GG_ = *β*_GC/CC_ = *β*_ALL_, see main text). Fasting plasma insulin levels are measured in pmol/l. Year of blood sample collection was included as a categorical confounding variable for both models. Sample sizes were 2358 and 2895 for insulin and T2D analyses, respectively.

### LD in the *PARL*/*ABCC5* Gene Region

**Figure 1:**
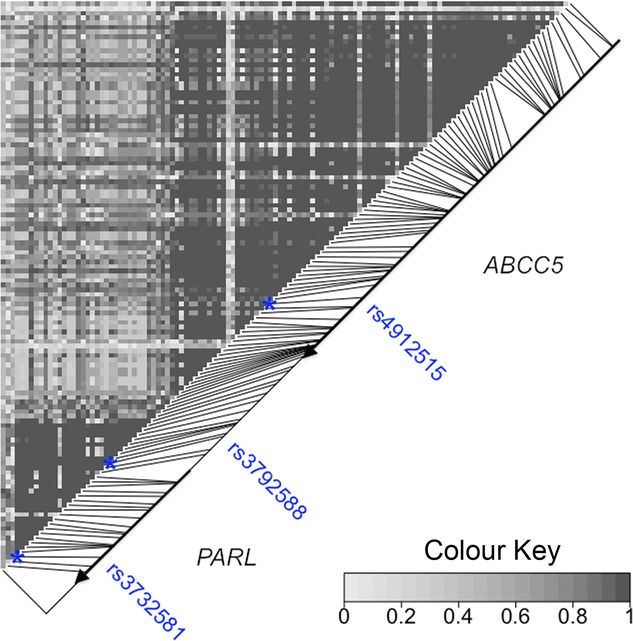
LD plot illustrating the degree of linkage disequilbrium in the *PARL*/*ABCC5* region. Pairwise marker LD (*D*') plotted for TwinsUK data for 122 SNPs in the 200 kb gene region (185,020–185,220 kb, Build 36). *PARL* includes SNP markers rs3732581 (Val262Leu, exon 7) and rs3792588 (promotor region) and *ABCC5* includes rs4912515 (intron 26, in closest proximity to the association model functional variant location estimate) with these three SNP locations highlighted as blue stars.

**Figure 2:**
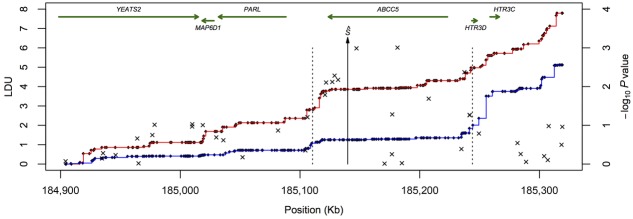
African and European genetic maps and scatter plot for WTCCC association between type 2 diabetes and single nucleotide polymorphisms. The graph presents a line plot for African (red) and European (blue) cumulative linkage disequilibria unit genetic maps (*Y*1-axis, LDU) and scatter plot for WTCCC type 2 diabetes association with SNPs in the *PARL*/*ABCC5* gene region (*Y*2-axis, −log_10_*P*). The genetic maps for HapMap Africans (ASW, Phase III) and Europeans (CEU, Phase II) are plotted with each dot representing an SNP location for the high-resolution HapMap samples from which these population-specific genetic maps are inferred. The arrowed vertical line labelled *Ŝ* represents the functional variant location estimate and the dotted lines, the 95% CI for the variant location. The total genetic distance (*Y*1-axis) for the same physical genomic region (*X*-axis) is greater for the African population compared to the European population, reflecting older ancestry and a greater number of historical recombination events for this population.

Figures 1 and 2 illustrate the high degree of LD within and moderate LD between the *PARL* and *ABCC5* genes. Figure 1 plots pairwise LD (*D*) and Figure 2 plots more informative fine scale genetic maps for HapMap European (CEU, European Americans) and African (ASW, African ancestry in Southwest USA) populations in the form of cumulative LDU (see Methods section). Note that while there is breakdown in LD between the two genes, there still remains some evidence of extended LD between the two. By plotting the genetic LDU maps against physical distance (kb), the nonlinear relationship is revealed as a “Block-Step” structure (See Fig. 2). “Blocks” of LD represent areas of low haplotype diversity, while “Steps” define LD breakdown, mainly caused by recombination. It is this high-resolution LD information that is used to locate potential functional variants associated with fasting insulin and glucose plasma levels and T2D for European and AA samples.

### The *PARL*/*ABCC5* Gene Region as a Candidate for Insulin Resistance and T2D

Table 2 presents the evidence for phenotypic association with genomic polymorphism data centred on the *PARL*/*ABCC5* gene region for European and AA populations. The WTCCC T2D sample provided significant evidence of association (*P* = 3E−06) with a functional variant location estimate *Ŝ* at 185,136 kb.

**Table 2:** Phenotype-genotype multi-marker association for *PARL*/*ABCC5* gene region in Europeans and African Americans

			*n*						
							
Data	Phenotype	Data	Sample	SNP	*P-*value	*Ŝ* (kb) location	95% CI		Gene (transcript strand)
Genomic	T2D	WTCCC	4864	83	3.E−06	**185,136**	185,108	185,215	intron 26 *ABCC5* (−)
Genomic	T2D	NIDDK	2004	104	0.01	**185,137**	185,114	185,218	intron 26 *ABCC5* (−)
Genomic	IGR	TwinsUK 610	2300	176	0.04	**185,136**	185,110	185,235	intron 26 *ABCC5* (−)
Genomic meta-analysis (3 samples):					1.E−07	**185,136**	185,108	185,235	intron 26 *ABCC5* (−)
Fat expression:	*ABCC5* mRNA	TwinsUK	700	202	1.E−11	**185,136**	185,109	185,235	intron 26 *ABCC5* (−)

All three samples show statistical evidence of association between T2D, IGR and *ABCC5* with a common causal variant estimated to most likely be located in intron 26. All samples provide exactly the same functional variant *Ŝ* location at or close to 185,136 kb (95% CI 185,108–185,235 kb, B36). Abbreviations: IGR, fasting insulin: glucose serum ratio levels, NIDDK, National Institute for Diabetes, Digestive and Kidney disease, T2D, type 2 diabetes, WTCCC, Wellcome Trust Case Control Consortium.

An identical location estimate for the functional variant was obtained for AA T2D case-control data with an *Ŝ* location at 185,137 kb with nominal significance (*P* = 0.01). The result was replicated for Europeans (TwinsUK) measured for IGR (*P* = 0.04) with the same location estimate as T2D at *ABCC5* intron 26 (185,136 kb). Meta-analysis of the three genomic result *P*-values (Table 2, WTCCC *P* = 3E−06, NIDDK *P* = 0.01 and TwinsUK 610K *P* = 0.04) using Fisher's method (Fisher, 1925), provided overall significant evidence of association (*P* = 3E−07) for a common functional variant located at 185,136 kb with 95% confidence intervals (CIs) for this location ranging from 185,108 to 185,235 kb. The CI includes the *ABCC5* gene, promoter and 3’ region (*ABCC5* gene co-ordinates: 185,120–185,215 kb), but excludes neighbouring *PARL*. The functional variant location estimate (arrowed line) and 95% CIs (dotted lines) are illustrated in Figure 2. This result implicates *ABCC5*, but also excludes the remaining 21 genes located within this gene rich region (see Methods for gene list) as being candidate disease susceptibility loci for T2D.

In relation to why previous GWA studies have not previously identified *ABCC5* as a T2D susceptibility gene, it is worth noting that while individual nominally significant SNP p-values nearest to the estimated *ABCC5* variant location at 185,136 kb for the WTCCC (e.g. rs3749441, *P* = 1E−03), NIDDK (e.g. rs1016752, *P* = 0.04) and TwinsUK samples (e.g. rs8180093, *P* = 0.002) do not pass genome-wide significance (*α* = 1E−05), when all the SNPs in the region are considered collectively as part of a multilocus model, they do provide strong evidence of association with T2D and insulin resistance (meta-analysis *P* = 3E−07). The two nominally significant WTCCC SNPs (marked ×) nearest the *Ŝ* location estimate in Figure 2 (arrow) are rs3749441 and rs3792582.

### eQTL Analyses

Table 2 presents the results for one adipose expression probe measured for the TwinsUK samples, which provides strong evidence of genetic association (*P* = 1E−011) with an identical location estimate *Ŝ* at *ABCC5* intron 26 (185,136 kb, 95% CI 185,109–185,235 kb). The remaining five adipose expression probes for *ABCC5* and *PARL* showed no evidence of eQTLs for the same analytical window (using threshold *α* = 1E−03, data not shown). This indicates that the associated functional variant in *ABCC5* is both associated with T2D and regulates *ABCC5* transcript expression.

Additional analyses show that the *ABCC5* Ilmn_1706531 probe for all tissues is consistently and significantly correlated with all three *PARL* probes (Table S2) indicating that *PARL* and *ABCC5* are co-expressed.

### Adipose *ABCC5* Expression is Positively Associated with Fasting Insulin, Visceral Fat Accumulation and T2D

We performed a series of analyses regressing T2D and T2D-related phenotypes upon each adipose probe in turn, using a mixed linear regression model to control for batch and age effects. Visceral fat was included as a T2D-related phenotype as this trait has previously been shown to be strongly associated with T2D and arguably may play a causal role in T2D disease onset (Direk et al., 2013). In particular, we were interested in adipose probe Ilmn_1706531 as this probe already showed evidence of eQTL genetic association with the longest *ABCC5* transcript (Table 2).

The univariate regression results (Table 3A) indicate that only *ABCC5* adipose probe Ilmn_1706531 was significantly associated with all phenotypes (HOMA2 *β* cell function, HOMA2 peripheral sensitivity, fasting IGR, visceral fat accumulation and T2D). Although *PARL* Ilmn_2257665 was also nominally associated with visceral fat (*P* = 0.04) and T2D (*P* = 0.01), these phenotypic associations appeared to be driven by *ABCC5* Ilmn_1706531 due to the observed negative correlation between the two probes (*r* = −0.38, Table S2). When HOMA2 sensitivity, visceral fat and T2D were regressed upon Ilmn_1706531, age, batch effects and the relevant nominally significant probes (Ilmn_2257665, Ilmn_1651964 and ILMN_2302358), only Ilmn_1706531 remained significantly and strongly associated with each phenotype (Table 3B). This confirmed that only adipose probe Ilmn_1706531 was independently associated with all five T2D-related phenotypes. The strength of association for this *ABCC5* probe (Table 3B) was estimated to have a correlation coefficient of +0.11 with HOMA2 *β* cell function (95% CI 0.04–0.17), −0.15 with HOMA2 peripheral sensitivity (95% CI −0.07 to −0.22), 0.11 with IGR (95% CI 0.05–0.18), a regression coefficient of 30 cm^2^ visceral fat per standard deviation (SD) increase in expression (95% CI 13.2–47.4; *r* = 0.19, 95% CI 0.12–0.17) and for T2D, an OR of 3.8 per SD increase in probe expression (95% CI 1.25–11.6). In addition, the prevalence of T2D is three times higher in subjects with high *ABCC5* expression compared to those with low expression (top vs. lowest expression quartile; 3% cf. 9%; *χ*^2^_3_ = 13, *P* = 0.005. However, note that for the 820 subjects with complete measured adipose expression data, only 37 individuals (4.5%) reported having T2D).

**Table 3:** Phenotypic association with adipose *PARL*/*ABCC5* gene expression

		HOMA *β* cell			HOMA sensitivity			Fasting IGR			Visceral fat			T2D		
						
Gene	Probe	Beta	SE	*P-*value	Beta	SE	*P-*value	Beta	SE	*P-*value	Beta	SE	*P-*value	Beta	SE	*P-*value
**A**																
*PARL*	ILMN_2341467	−0.03	0.16	0.84	0.03	0.17	0.86	−0.01	0.18	0.96	−1.8	12.1	0.88	−0.71	1.02	0.48
*PARL*	ILMN_1731354	−0.04	0.16	0.81	0.01	0.17	0.96	0.06	0.18	0.73	−13.9	12.8	0.28	−0.52	1.13	0.65
*PARL*	ILMN_2257665	0.07	0.08	0.44	−0.06	0.09	0.51	−0.04	0.10	0.67	−15.1	7.5	0.04	−1.67	0.70	0.02
*ABCC5*	ILMN_1706531	0.27	0.09	0.001	−0.38	0.10	2.E−04	0.38	0.10	1.E−04	33.8	6.9	1.E−06	1.67	0.44	2.E−04
*ABCC5*	ILMN_1651964	0.24	0.16	0.13	−0.27	0.15	0.07	0.30	0.14	0.03	30.7	9.7	1.E−03	1.82	0.73	0.01
*ABCC5*	ILMN_2302358	0.54	0.36	0.14	−0.71	0.33	0.03	0.25	0.31	0.40	−5.9	20.1	0.77	2.25	1.67	0.18
**B**																
*PARL*	ILMN_2257665	–	–	–	–	–	–	–	–	–	−1.3	8.0	0.88	0.14	0.47	0.77
*ABCC5*	ILMN_1706531	0.27	0.09	0.001	−0.41	0.13	0.001	0.37	0.12	2.E−03	30.3	8.7	5.E−04	1.34	0.43	2.E−03
*ABCC5*	ILMN_1651964	–	–	–	0.04	0.18	0.83	0.03	0.16	0.84	7.8	11.4	0.50	−0.61	1.03	0.55
*ABCC5*	ILMN_2302358	–	–	–	−0.65	0.33	0.05	–	–	–	–	–	–	–	–	–

Only adipose probe Ilmn_1706531 is associated with all phenotypes in univariate (A) and multiple regression analyses (B). Transcript analysis sample sizes for HOMA *β* cell, HOMA sensitivity, IGR, visceral fat and T2D were 680, 680, 680, 619 and 820, respectively. HOMA and fasting IGR measures are quantile normalised. Visceral fat is body area in cm^2^, while risk of T2D is on the logit scale, with exp(1.34) equal to an odds ratio of 3.8 (95% CI 1.25–11.6).

The association between IGR and adipose probe Ilmn_1706531 was largely driven by fasting insulin (*P* = 1E−04) rather than glucose levels (*P* = 0.02) with raised adipose *ABCC5* transcript levels associated with increased *β* cell function (Homeostasis Model Assessment HOMA2B, *P* = 1E−03) and reduced peripheral insulin sensitivity (HOMA2S, *P* = 2E−04). Significantly, none of the mRNA probes for LCL and skin tissues—including *ABCC5* probe Ilmn_1706531—were associated with phenotypes IGR, visceral fat or T2D (data not shown).

## Discussion

**Figure 3:**
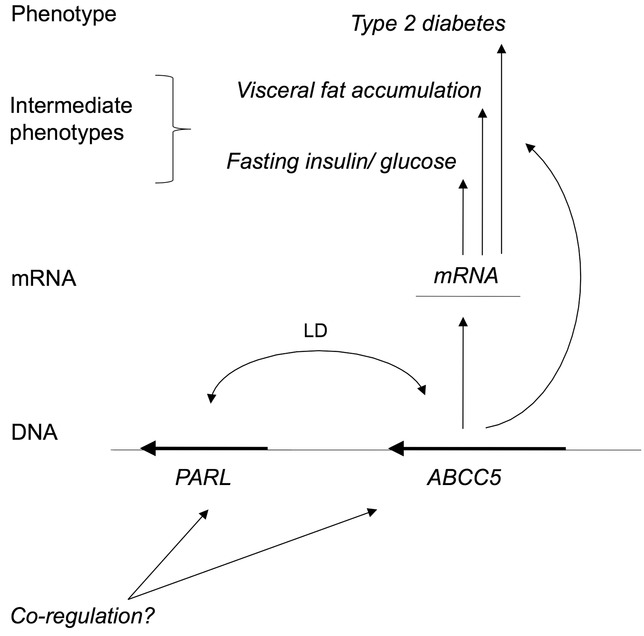
Diagram illustrating *ABCC5* genetic and mRNA association with intermediate phenotypes and type 2 diabetes. An eQTL in *ABCC5* (intron 26) regulates mRNA expression levels and is associated with fasting plasma insulin and glucose levels, visceral fat accumulation and T2D for TwinsUK data. *ABCC5* transcript Ilmn_1706531 is also strongly associated with intermediate phenotypes and T2D (see Table 3), suggesting the causal mechanism of association is mediated via mRNA expression that are tissue and transcript specific. Double-headed arrows indicate correlation and curved lines association, but are not directly causal.

In this study, we confirm that the nonsynonymous *PARL* SNP rs3732581 (exon 7) and the *PARL* gene itself, show no evidence of association with fasting plasma insulin and glucose levels or T2D for European and AA samples. By contrast however, for the first time we demonstrate that the neighbouring *ABCC5* transporter gene shows replicated evidence of genetic association with T2D and insulin resistance at the same locus for three populations. The same variant observed to be associated with T2D also plays a regulatory role in controlling *ABCC5* adipose expression levels, with elevated *ABCC5* expression levels in turn significantly associated with reduced insulin peripheral sensitivity in nondiabetic individuals (*r* = −0.15), increased visceral fat accumulation (*r* = 0.19) and increased risk of T2D (OR = 3.8, 95% CI 1.25–11.6), all of which indicates that over expression of *ABCC5* may be causally implicated in T2D pathophysiology (see Fig. 3). By contrast, *PARL* genetic variants and gene expression are not associated with intermediate phenotypes or T2D for human data, suggesting that previously observed animal and experimental data for association between *PARL* dysfunction and T2D may be a consequence rather than a cause of disease onset.

The *ABCC5* genetic variant may also explain contradictory human association results observed for Val262Leu, if previously observed association with *PARL* is in fact due to confounding LD with functional variants in *ABCC5*. For example, SNPs such as rs4912515 (Fig. 1)—in immediate proximity to the *ABCC5* variant *Ŝ* location estimate—show significant evidence of long-range LD with Val262Leu in *PARL*.

There are three major strengths to the genetic map–based test of association that should be mentioned here. The first is that this elegant model is highly interpretable, both in terms of population genetic theory (Malécot, 1948) and in the context of association mapping (Maniatis et al., 2002; Wellcome Trust Case Control Consortium, 2007). The second is that the model is more sensitive than single SNP tests, which are widely used in GWA studies, to detect functional variants that are potentially associated with a combination of neighbouring genotyped markers, but not necessarily in high LD with any single genotyped marker. For the commercial SNP arrays that are currently available [and when the assumption of high allelic identity begins to breakdown (Pritchard & Cox, 2002)], a multimarker approach provides a much more realistic scenario than the current practice of assuming a putative functional variant is in high LD with just one genotyped marker. Direct estimation of the functional variant location (along with a location 95% CI) provides more precise location inferences than individual associated SNPs and provides a considerable degree of commensurability between different commercial genotyping platforms that other methods do not. Thirdly, gene mapping is far more efficient using markers located upon a genetic rather than a physical map (Maniatis, 2007).

How does the evidence from previous animal model and functional studies for reduced *PARL* expression associated with observed and induced T2D (Walder et al., 2005; Tang et al., 2009; Civitarese et al., 2010), square with current results for observed human genomic and expression data implicating *ABCC5*, but not *PARL*? We believe the most likely explanation for these observations is that *ABCC5* plays a contributory causal role in T2D onset, while *PARL* dysfunction appears to be a consequence rather than a cause of insulin resistance and disease state. This view is supported by (1) evidence for a functional genetic variant in *ABCC5* associated with T2D in multiple human populations that also regulates *ABCC5* expression and (2) empirically, *PARL* and *ABCC5* mRNA transcripts appear to be co-expressed, perhaps suggesting co-regulation of the two genes. *ABCC5* may also (partly) drive the expression of *PARL*, since the observed association between phenotype (T2D, visceral fat accumulation) and *PARL* expression (Ilmn_2257665, Table 3) is conditionally independent, once *ABCC5* expression (Ilmn_1706531) is accounted for.

*ABCC5* is a member of the ATP-binding cassette transporter superfamily (Dean & Allikmets, 2001), transporting cyclic nucleotides such as cyclic guanosine monophosphate (cGMP) (Jedlitschky et al., 2000), where cGMP is a common regulator of ion channel conductance, glycogenolysis and cellular apoptosis. Although ABC transporter genes including *ABCC5* have been studied in relation to cancer treatment and drug resistance (Wijnholds et al., 2000; Tamaki et al., 2011), little is known about *ABCC5* in relation to T2D. One exception to this is an ABC transporter expression study of uptake and efflux transporters in the liver of rats with induced diabetes (streptozotocin injection). The study demonstrates that liver ABCC5 (MRP5) protein expression levels (Western blot) in diabetic rats fed high-fat diets collapse (to 4%) when compared to control rat liver expression levels (Nowicki et al., 2008).

This study supports the transporter gene superfamily as worthy of further investigation in relation to common T2D (Xiang & Xiao-Dong, 2009), with the subfamilies of ABCC and ABCG already implicated in different ways (Matsuo, 2010). Mutations in the sulfonylurea receptor (SUR) 1 encoded by *ABCC8* have previously been shown to cause neonatal diabetes (Greeley et al., 2011), maturity onset diabetes of the young (MODY) (Bowman et al., 2012) and adult T2D (Tarasov et al., 2008). In addition, the ABCG transporter genes have also been implicated in cholesterol transport in animal models of T2D (Levy et al., 2010).

A key question raised by this study is the nature of any potential functional relationship between *PARL* and *ABCC5*. Future work will need to include efforts to identify the functional variant (for example a regulatory element in intron 26) and undertake further functional studies such as cell culture work to more clearly establish the molecular mechanisms by which *ABCC5* confers risk of T2D.

In conclusion, the *ABCC5* polymorphism is associated with T2D in three populations of European and African ancestry and is observed to regulate *ABCC5* expression levels in healthy middle-aged Europeans. *ABCC5* expression levels are in turn associated with insulin resistance, visceral fat accumulation and disease. The fact that ABCC5 is a transporter protein with *ABCC* transporters prioritised for drug research (Hillgren et al., 2013) may also provide additional opportunities for drug target development in the treatment of T2D.

## Supporting Information

Disclaimer: Supplementary materials have been peer-reviewed but not copyedited.

**Table S1** Transcript expression probes for *PARL* and *ABCC5*. **Table S2** TwinsUK transcript expression correlation structure for *PARL* and *ABCC5*.
