# Supplementary material for: ABCC5 Transporter is a Novel Type 2 Diabetes Susceptibility Gene in European and African American Populations
Source: Ann Hum Genet. 2014 Aug 12;78(5):333–44. doi: 10.1111/ahg.12072 (PMC4173130; doi:10.1111/ahg.12072)
Supplement: Supplementary file 1 [file ahg0078-0333-SD1.doc]

**Table S1**. **Transcript expression probes for *PARL* and *ABCC5*.** The *PARL* probes Ilmn_2341467 and Ilmn_1731354 both mark the same seven *PARL* mRNA transcripts, while Ilmn_2257665 marks two additional *PARL* transcripts. The three *ABCC5* probes mark three, four and nine *ABCC5* mRNA transcripts, respectively. Probe ILMN_1706531 marks two full-length transcripts (ABCC5-001 and ABCC5-201) that both include intron 26.

**Table S2: TwinsUK transcript expression correlation structure for *PARL* and *ABCC5*.** The pearson product moment correlation is presented between six mRNA expression probes for *PARL* and *ABCC5* measured in adipose, LCL and skin tissue samples. The transcript correlations are for probes within the same gene, while highlighted areas refer to between-gene correlations (for the same tissue). The residuals for batch effects were first taken for each probe before correlation coefficients were estimated to provide a partial correlation coefficient controlling for batch effect.
